# Eplerenone restores 24-h blood pressure circadian rhythm and reduces advanced glycation end-products in rhesus macaques with spontaneous hypertensive metabolic syndrome

**DOI:** 10.1038/srep23957

**Published:** 2016-04-01

**Authors:** Yan Zhang, Wen Zheng, Yuli Liu, Jue Wang, Ying Peng, Haibao Shang, Ning Hou, Xiaomin Hu, Yi Ding, Yao Xiao, Can Wang, Fanxin Zeng, Jiaming Mao, Jun Zhang, Dongwei Ma, Xueting Sun, Chuanyun Li, Rui-Ping Xiao, Xiuqin Zhang

**Affiliations:** 1 State Key Laboratory of Membrane Biology, Institute of Molecular Medicine, Peking University, Beijing 100871, China; 2 Peking-Tsinghua Center for Life Sciences, Beijing 100871, China; 3 Beijing Key Laboratory of Cardiometabolic Molecular Medicine, Peking University, Beijing 100871, China; 4 Department of Surgery, Third Affiliated Hospital of Peking University, Beijing 100871, China

## Abstract

Hypertension is often associated with metabolic syndrome (MetS), and serves as a risk factor of MetS and its complications. Blood pressure circadian rhythm in hypertensive patients has been suggested to contribute to cardiovascular consequences and organ damage of hypertension. But circadian changes of BP and their response to drugs have not been clearly investigated in non-human primates (NHPs) of MetS with hypertension. Here, we identified 16 elderly, hypertensive MetS rhesus monkeys from our in-house cohort. With implanted telemetry, we investigate BP changes and its circadian rhythm, together with the effect of antihypertensive drugs on BP and its diurnal fluctuation. MetS hypertensive monkeys displayed higher BP, obesity, glucose intolerance, and dyslipidemia. We also confirmed impaired 24-h BP circadian rhythm in MetS hypertensive monkeys. Importantly, Eplerenone, a mineralocorticoid receptor blocker, exerts multiple beneficial effects in MetS hypertensive monkeys, including BP reduction, 24-h BP circadian rhythm restoration, and decreased plasma concentration of inflammation factors and advanced glycation end-products. In summary, we identified a naturally-developed hypertensive MetS NHP model, which is of great value in the studies on pathogenesis of MetS-associated hypertension and development of novel therapeutic strategies. We also provided multiple novel mechanistic insights of the beneficial effect of Eplerenone on MetS with hypertension.

Hypertension is frequently present in patients with metabolic syndrome (MetS) and an important risk factor for cardiovascular-related morbidity and mortality^1^,^2^. Both insulin resistance (IR) and obesity elevate blood pressure (BP) *via* increased oxidative stress, inflammation, salt retention, and impaired generation of nitric oxide^3^. Hypertensive patients with MetS have severe IR, together with elevated blood adipokines, such as leptin, tumor necrosis factor-α (TNF-α), interleukin-6 (IL-6), and resistin, which not only over-activate the sympathetic nervous system and rennin-angiotensin-aldosterone system, but also promote inflammation, endothelial dysfunction, and atherosclerosis^4^,^5^,^6^. Collectively, these factors contribute to the worsening of hypertension and significantly increase cardiovascular morbidity and mortality.

In the recently-published Guideline for Managing BP, thiazide-type diuretics, Ca^2+^ channel blockers (CCBs), angiotensin-converting enzyme inhibitors (ACEIs), and angiotensin receptor blockers (ARBs) are recommended as first-line drugs for the treatment of hypertension^7^. However, the use of α- or β-blockers as an initial treatment is controversial^8^,^9^,^10^. ACEIs, ARBs, and CCBs have beneficial effects on MetS by reducing inflammation^11^, increasing insulin sensitivity^12^, and improving the secretion of adiponectin^13^. It has been shown that another class of anti-hypertensive drugs, the mineralocorticoid receptor (MR) blockers, have greater BP lowering effects and cardiovascular benefits^14^,^15^,^16^,^17^.

The cardiovascular system exhibits a distinct temporal organization, particularly with respect to diurnal variations in BP and heart rate (HR)^18^. Arterial BP has a circadian variation, usually expressed as a 10–20% drop during the night, and a rapid rise upon awakening. This pattern is typically disturbed in hypertensive patients^19^. Several lines of evidence suggest that, in addition to contributing to elevated BP in cardiovascular outcomes, disturbed 24-h BP circadian rhythm is associated with increased incidence of cardiovascular events and mortality^20^. These studies indicate that it is important to manage the circadian rhythm of BP in hypertension therapy^21^,^22^. There is growing interest in how current and newly-discovered medications that lower BP affect the circadian pattern^23^.

As a consequence of their phylogenetic proximity to humans, non-human primates (NHPs) are particularly relevant species for preclinical studies. Prior studies have shown that NHPs naturally develop symptoms of MetS and hypertension similar to those in humans^24^,^25^. However, their innate aggressiveness and the difficulty of training NHPs make it hard to measure cardiovascular parameters under conscious and freely-moving conditions. Most examinations have to be performed under anesthesia or restraint, which not only affect BP, but also make it impossible to assess the circadian rhythm. Therefore, very few studies on the circadian BP rhythm are available, other than those in normal marmosets that show a circadian rhythm similar to humans^26^,^27^,^28^. To date, no data are available for other NHPs, and in particular there are no comparative data from normotensive and hypertensive NHPs, due to the lack of suitable measurement devices and a well-characterized stable population of animals.

In the current study, we established a NHP model with both MetS and hypertension. And with an implanted telemetry system to continuously record BP, we tested the BP-lowering effects and the restoration of impaired 24-h BP circadian rhythm by Eplerenone (an MR blocker) in the MetS hypertensive NHP model. We also accessed the plasma concentration of inflammation factors and advanced glycation end-products before and after Eplerenone treatment. This study not only provides a valuable tool for assessment of the pathogenesis and pharmacological effects in hypertension in NHPs with MetS, but also sheds light on the novel therapeutic mechanisms of MR antagonists.

## Results

### Identification and characterization of naturally-developed hypertension in rhesus monkeys with spontaneous MetS

We have reported previously that rhesus monkeys develop typical spontaneous MetS with aging^25^. Among the 5 MetS parameters, high BP, especially elevated Systolic BP (SBP), presents most frequently^25^. According to the hypertension-diagnosis standard for humans and NHPs^29^, we set SBP ≥140 mmHg or diastolic BP (DBP) ≥90 mmHg (average of the last two in-house tests under ketamine anesthesia) as the criteria for hypertension in the monkeys, and SBP <130 mmHg/DBP <90 mmHg as normotension. Based on these criteria, from the continuous follow-up data of our previous reported cohort monkeys and other in-house cohort of monkeys, we identified 17 control normotensive (out of 23 control monkeys) and 16 MetS hypertensive monkeys (out of 23 MetS monkeys that did not yet have type 2 diabetes).

**Figure 1:**
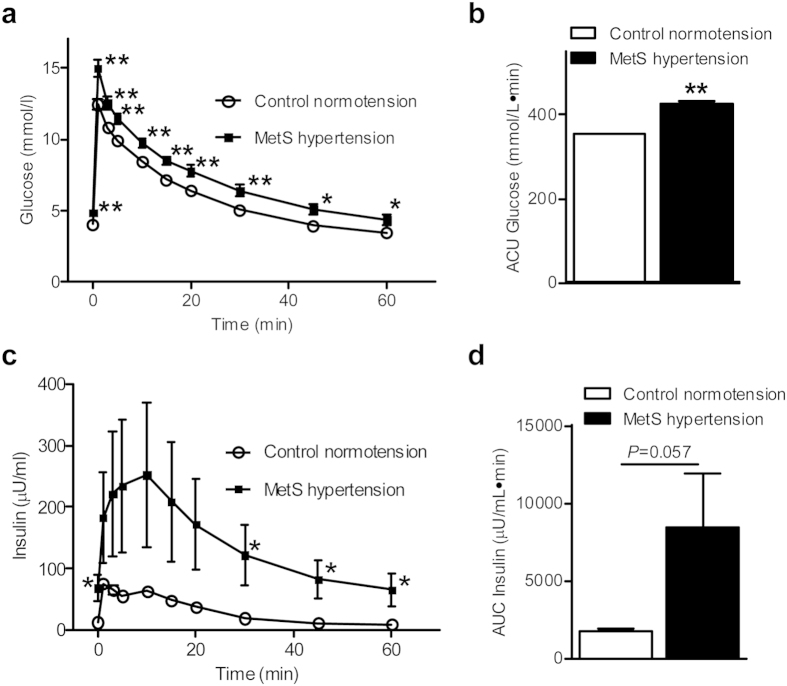
Plasma glucose and insulin responses during the intravenous glucose tolerance test. **(a)** Glucose excursion. **(b)** Area under the glucose curve. **(c)** Insulin excursion. **(d)** Area under the insulin curve. Data are expressed as mean ± SEM; n = 17 for control normotensive group and 16 for MetS hypertensive group. *P < 0.05, **P < 0.01 *vs* corresponding control normotension group. MetS, metabolic syndrome.

The metabolic and anthropometric characteristics of control normotensive and MetS hypertensive monkeys from the latest two tests are summarized in Table 1. Compared with the normotensive group, MetS hypertensive monkeys displayed significantly higher SBP and DBP, together with increased waist circumference, body weight, triglycerides, fasting plasma glucose, fasting insulin, homeostasis model assessment-IR, and C-reactive protein. High-density lipoprotein was lower in MetS hypertensive monkeys. However, low-density lipoprotein and total cholesterol were similar in both groups. Plasma glucose levels were significantly higher in MetS hypertensive monkeys than in control normotensive monkeys at 1, 3, 5, 10, 15, 20, 30, 45, and 60 min after glucose challenge (Fig. 1a). Similarly, the area under the curve (AUC) was significantly greater in MetS hypertensive monkeys than in controls (Fig. 1b). The rise in insulin levels induced by glucose and the corresponding AUC showed an increasing trend in the MetS hypertensive group (Fig. 1c,d). Collectively, the data indicate that hypertension is associated with obesity, dyslipidemia, and IR in MetS hypertensive monkeys.

### BP under ketamine anesthesia was comparable to that measured via chronic, implanted telemetry

During follow-up screening, the BP and HR were measured under ketamine anesthesia. To compare the BP level under anesthesia and in freely-moving conditions, and to further obtain accurate 24-h BP in conscious monkeys, we implanted the DSI telemetry system into 5 control normotensive and 8 MetS hypertensive monkeys (one of the MetS monkeys was excluded due to a technical problem).

**Figure 2:**
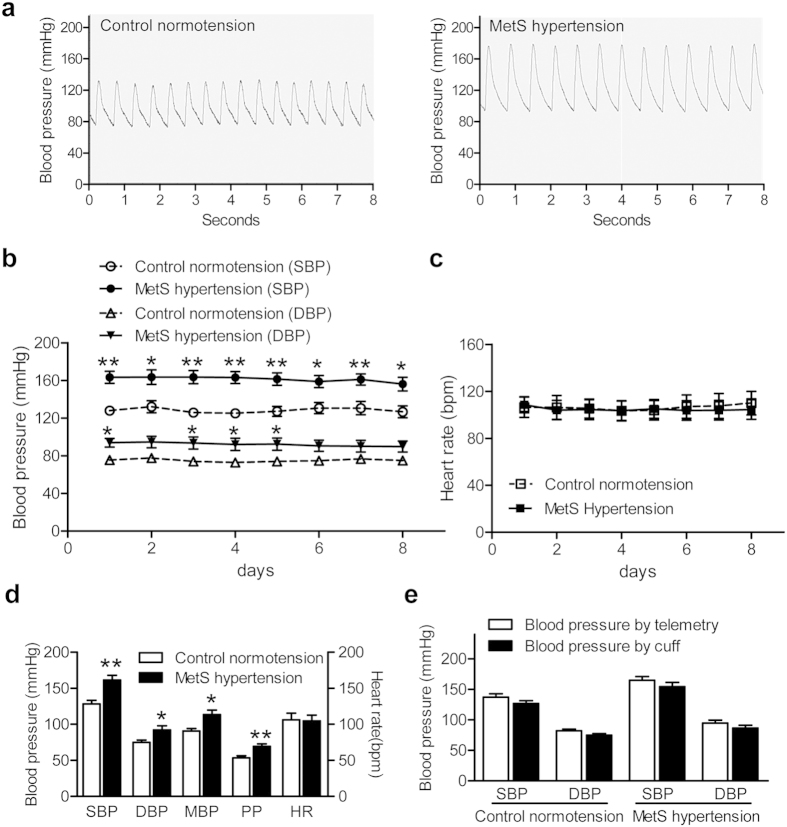
BP recorded by telemetry in control normotensive and MetS hypertensive groups. **(a)** Representative waveforms of BP measured by telemetry in control normotensive (left panel) and MetS hypertensive (right panel) monkeys. **(b,c)** 24-h averaged SBP and DBP **(b)** and HR **(c)** of control normotensive (n = 5) and MetS hypertensive groups (n = 7). **(d)** Averaged SBP, DBP, MBP, PP, and HR during the 8 days in panels B and C in control normotensive (n = 5) and MetS hypertensive (n = 7) monkeys (*P < 0.05, **P < 0.01 *vs* control normotension). **(e)** Comparison of SBP and DBP recorded by telemetry (09:00–12:00) and cuff (during screening) in control normotensive (n = 5) and MetS hypertensive (n = 7) monkeys. Data are expressed as mean ± SEM. SBP, systolic blood pressure; DBP, diastolic blood pressure; MBP, mean blood pressure; PP, pulse pressure; HR, heart rate; MetS, metabolic syndrome.

The arterial BP waveforms from both groups recorded by telemetry are shown in Fig. 2a. After 2 weeks of recovery from surgery, we confirmed that the 24-h average BP (SBP and DBP) and HR were stable in both the control normotensive and MetS hypertensive groups throughout 8 continuous days of recording (Fig. 2b,c). The 24-h average values (mean of 8 days) in the MetS hypertensive monkeys were 161.0 ± 6.5 (SBP), 92.1 ± 5.9 (DBP), 113.5 ± 6.3 (mean BP, MBP), and 69.3 ± 3.3 mmHg (pulse pressure, PP), all of which were higher (*P* < 0.01 for SBP and PP, *P* < 0.05 for DBP and MBP) than those of the control group (128 ± 4.9, 74.9 ± 3.1, 90.7 ± 3.4, and 53.3 ± 3.0 mmHg) (Fig. 2d). HR was similar in the 2 groups (Fig. 2d), which differed from the results during anesthesia (Table 1). This may be attributed to different responses of the 2 groups of monkeys to anesthesia. We also measured BP with a cuff in anesthetized monkeys and compared the values with those obtained from the telemetry system at the same time and found a close correlation (Supplemental Fig. 1), suggesting that both of methods are suitable in monkeys.

In our previous tests, the BP and HR measurements under anesthesia were all performed between 09:00 and 12:00. Since BP and HR have a diurnal variation, we compared the average BP and HR from our last two tests with the 4-day average recorded by telemetry between 09:00 and 12:00. Although the SBP and DBP recorded in the latest 2 tests under anesthesia were slightly lower than those recorded in conscious animals by telemetry in both the control normotensive and MetS hypertensive groups, the differences were not significant (Fig. 2e). These data indicate that repeated BP measurement with well-controlled anesthesia is a convenient and reliable method to identify hypertensive animals from a population of NHPs.

### Telemetry and model validation with CCB treatment

**Figure 3:**
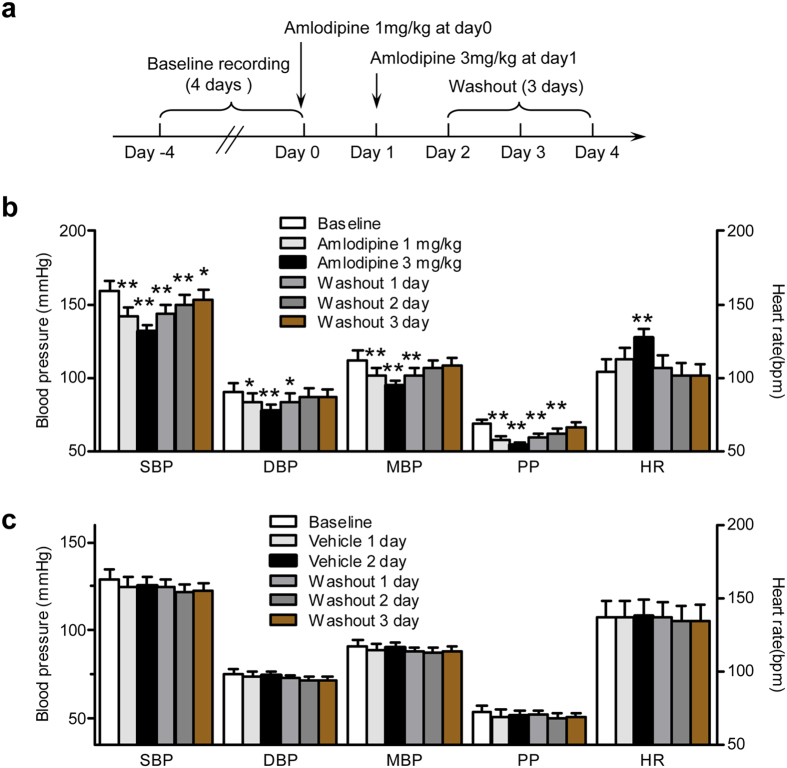
Effect of Amlodipine on MetS hypertensive monkeys. **(a)** Protocol for Amlodipine treatment in MetS hypertensive monkeys. **(b,c)** SBP, DBP, MBP, PP, and HR of control normotensive (n = 5) and MetS hypertensive (n = 7) monkeys. MetS hypertensive monkeys **(b)** were treated with Amlodipine. Control normotensive monkeys **(c)** were treated with vehicle during the same periods (*P < 0.05, **P < 0.01 *vs* baseline values of the corresponding group). Data are expressed as mean ± SEM. SBP, systolic blood pressure; DBP, diastolic blood pressure; MBP, mean blood pressure; PP, pulse pressure; HR, heart rate; MetS, metabolic syndrome.

To ensure that the implanted BP transmitters were functional and able to detect changes in BP, after baseline recording for 4 days, Amlodipine, a long-acting CCB, was orally delivered to the MetS hypertensive monkeys (Fig. 3a). Control normotensive animals received vehicle during the same period. Consistent with clinical studies^30^, the SBP, DBP, MBP, and PP of these monkeys were significantly decreased upon Amlodipine treatment in a dose-dependent manner, while HR was elevated (Fig. 3b). All of these indices returned to baseline by 3 days after Amlodipine washout (Fig. 3b). The SBP, DBP, MBP, PP, and HR of the control normotensive monkeys, which received vehicle, remained unchanged (Fig. 3c), further confirming the capacity of Amlodipine to lower BP and the stability of the telemetry recording.

### 24-h BP circadian rhythm in rhesus monkeys

Taking advantage of the MetS hypertensive model and the telemetry system, we analyzed the 24-h BP circadian rhythm in the control normotensive and the MetS hypertensive rhesus monkeys. The BP values used to analyze circadian rhythms in both groups of monkeys were the average of 4 days of baseline recording.

**Figure 4:**
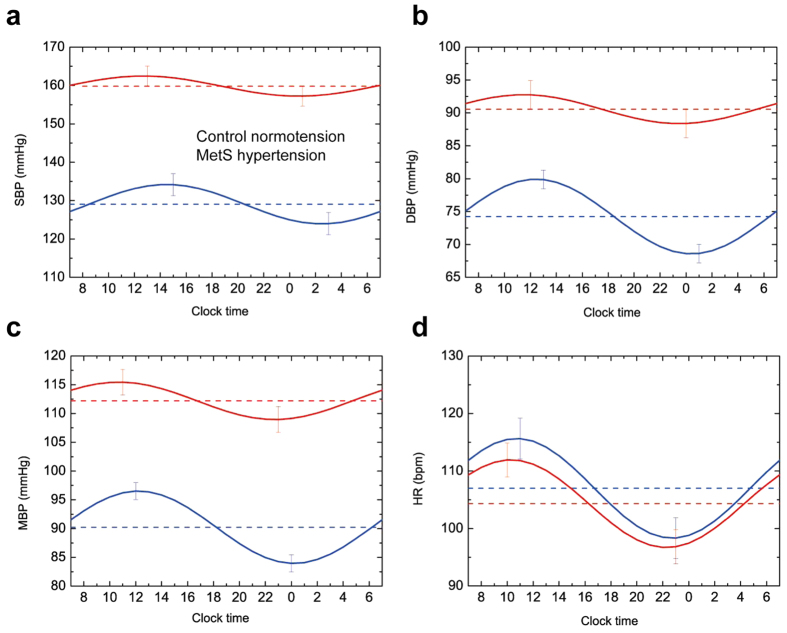
Circadian rhythm of blood pressure in control normotensive and MetS hypertensive groups. **(a–d)** Circadian rhythms of SBP **(a)**, DBP **(b)**, MBP **(c)**, and HR **(d)** of control normotension (n = 5) and MetS hypertensive groups (n = 7). SBP, DBP, MBP and HR were continuously collected as the average of every 20 seconds with the telemetry system. Error bars show the 95% confidence intervals of the amplitude. Data are expressed as mean ± SEM. SBP indicates systolic blood pressure; DBP, diastolic blood pressure; MBP, mean blood pressure; HR, heart rate; MetS, metabolic syndrome.

The pattern of BP and HR fluctuations over 24 h was similar in both control normotensive and MetS hypertensive monkeys, *i.e.* higher during the day and lower at night, but the BP variability was decreased in the MetS hypertensive group (Fig. 4a–c, Table 2), consistent with the findings in humans^19^,^31^. The amplitudes of SBP, DBP, and MBP in the MetS hypertensive group were lower than those in control normotensive monkeys, although only DBP reached significance (*P* = 0.007) (Table 2). The HR amplitude was similar in the 2 groups (Fig. 4d, Table 2). Overall, these results provide a valuable reference for using rhesus monkeys as a model in which to perform detailed chronobiological cardiovascular studies, and for preclinical pharmacological tests on BP control and circadian rhythm.

### Effect of MR blocker on hypertensive MetS monkeys

**Figure 5:**
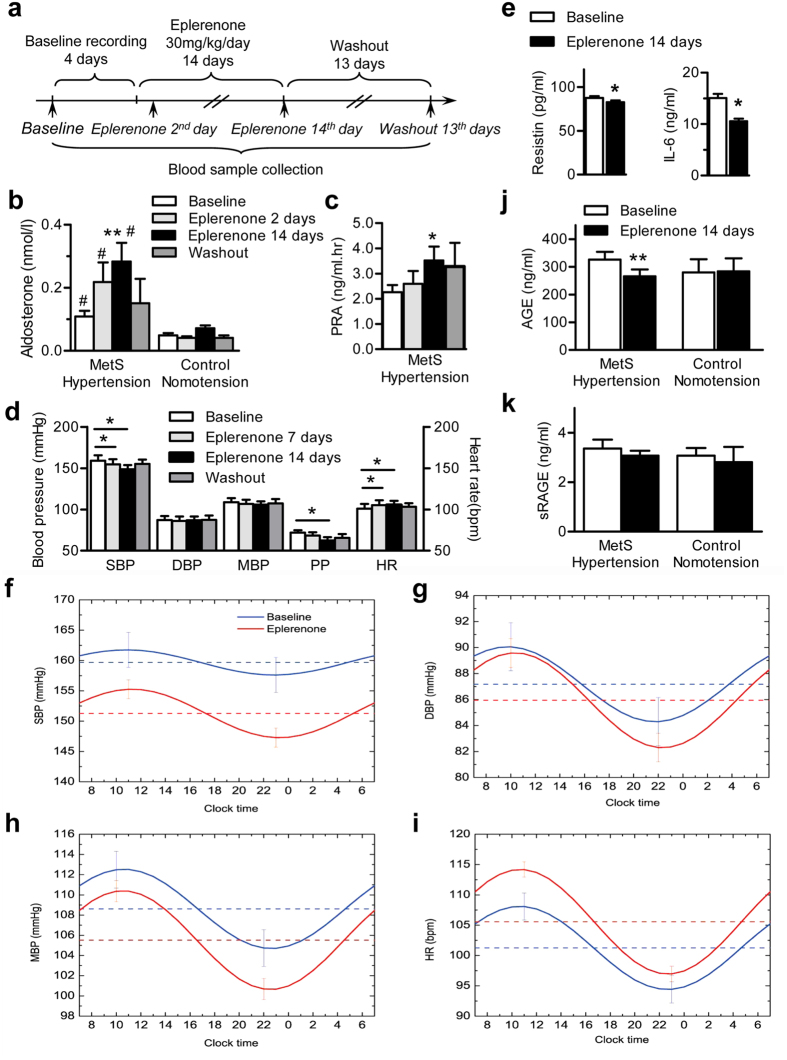
Effect of Eplerenone on MetS hypertensive monkeys. **(a)** Protocol for Eplerenone treatment. **(b)** Plasma concentrations of aldosterone of MetS hypertensive (n = 7) and control normotensive (n = 5) monkeys during baseline, Eplerenone (for MetS hypertensive) (30 mg/kg/day, *p.o.*, once a day) or Vehicle (for control normotensive) treatment for 2 days and 2 weeks, and washout for 13 days. **(c)** PRA of MetS hypertensive (n = 7) monkeys during baseline and Eplerenone treatment (30 mg/kg/day, *p.o.*, once a day) for 14 days and washout for 13 days. **(d)** SBP, DBP, MBP, PP, and HR in MetS hypertensive monkeys at baseline and after periods of Eplerenone treatment (n = 7). **(e)** Plasma concentrations of resistin (left panel) and IL-6 (right panel) of MetS hypertensive monkeys at baseline and after 14 days of Eplerenone treatment (*P < 0.05). **(f–i)** Comparison of 24-h circadian rhythms of SBP **(f)**, DBP **(g)**, MBP **(h)**, and HR **(i)** in MetS hypertensive monkeys before and after Eplerenone treatment (n = 7). The error bars in (**f–i)** show the 95% confidence interval of the amplitude. **(j,k)** Plasma concentrations of AGE **(j)** and sRAGE **(k)** of Control nomotension and MetS hypertensive monkeys at baseline and after 14 days of Eplerenone treatment (*P < 0.05 *vs* baseline values of the corresponding group). Data are expressed as mean ± SEM. For panels (**b,c),** *P < 0.05, **P < 0.01 *vs* baseline, ^#^*P* < 0.05 vs the corresponding group values of control normotension. MetS, metabolic syndrome; PRA, plasma rennin activity; SBP, systolic blood pressure; DBP, diastolic blood pressure; MBP, mean blood pressure; HR, heart rate; AGE, advanced glycation end-products, sRAGE, soluble receptor of AGE.

The effect of Eplerenone, a selective MR antagonist, on BP and BP circadian rhythm was evaluated in the MetS hypertensive animals. Baseline measurements of BP and HR were recorded for 4 days, Eplerenone was then administered. During treatment, the monkeys were anesthetized to collect blood samples at baseline, 1, and 14 days after Eplerenone treatment and at 13 days after washout (Fig. 5a). Consistent with clinical reports^32^, the plasma aldosterone level in MetS hypertensive monkeys was significantly higher than control monkeys, and it was further increased after administration of eplerenone by compensation (Fig. 5b). Similarly, plasma renin activity (PRA) also increased significantly after eplerenone treatment (Fig. 5c). In the monkeys that received Eplerenone, the SBP significantly decreased during weeks 1 and 2 of treatment compared with baseline (154.6 ± 6.4 and 148.1 ± 5.7 *vs* 159.1 ± 6.4 mmHg, *P* < 0.05) (Fig. 5d). PP was lower than baseline during week 2 of treatment (61.7 ± 4.8 *vs* 72.0 ± 3.0 mmHg, *P* < 0.05) (Fig. 5d). HR was elevated 2 weeks after treatment, while MBP and DBP remained unchanged (Fig. 5d). Eplerenone treatment for 2 weeks altered neither plasma potassium and lipid, nor hepatic and kidney function (Supplemental Fig. 2).

Besides its BP-lowering effect, we found that Eplerenone also significantly decreased the plasma concentrations of resistin and IL-6 (Fig. 5e), both of which have been associated with inflammation and hypertension in obesity^33^. Inhibiting resistin and IL-6, together with inflammation, may also contribute to the cardiac beneficial effect of Eplerenone as anti-hypertensive therapy. However, the other adipokines (adiponectin, leptin, monocyte chemoattractant protein-1 (MCP-1), and TNF-α) were not changed by Eplerenone treatment in our experiments (Supplemental Fig. 3).

Several recent studies have investigated the relationship between BP circadian rhythm and cardiac outcome, and have drawn diverse conclusions depending on treatments and populations in different studies^21^,^31^,^34^. And so far, there is no data on the role of Eplerenone in the regulation of 24-h BP circadian rhythm. In this study, we tracked the rhythmicity of diurnal BP patterns before and after Eplerenone treatment. The diurnal variations of SBP, DBP, and MBP (indicated by the amplitude) were restored in MetS hypertension monkeys without changing the BP diurnal fluctuation after 2 weeks of treatment, although only that of MBP reached significance (*P* = 0.004) (Fig. 5f–h, Table 3). The HR amplitude was slightly increased with treatment (Fig. 5i, Table 3). Restoring the pattern of a nocturnal decrease in BP may also contribute to the cardiac beneficial effects of Eplerenone in hypertensive patients.

Advanced glycation end-products (AGEs) have been established to play a key role in the development and progression of multiples cardiovascular diseases and metabolic disorders. Although MR antagonists have been shown to perform beneficial effects on the patients with hypertension and MetS, so far, there is no report on the relationship between MR antagonists and AGEs. We found that 2-week Eplerenone treatment significantly decreased the plasma AGE concentration in MetS hypertensive monkeys to a similar level as in control normotensive ones (Fig. 5j). And Eplerenone treatment did not affect the plasma concentration of soluble receptor of AGE (sRAGE), which inhibits AGE signaling via targeting the receptor of AGEs (RAGE) (Fig. 5k). These data underlie a correlation between MR antagonists and AGE system.

## Discussion

In recent decades, unhealthy lifestyles characterized by sedentary behavior and a high intake of calories have driven a burst in the prevalence of MetS. Hypertension presents most frequently within this clinical constellation^1^,^2^, and is a critical risk factor for cardiovascular complications and other organ damage. Understanding the pathogenesis will provide novel or more effective therapeutic strategies for hypertension. In the present study, we devised, implemented, and validated a set of strategies to establish a natural model of MetS hypertensive rhesus monkeys. We also pursued to elucidate the mechanisms of the beneficial effects of MR blockers, a newly identified anti-hypertensive drug proved to benefit the metabolic disorders.

Hypertension associated with MetS has unique pathophysiological characteristics that provide clinical challenges for both mechanistic studies and successful therapeutic interventions. MetS-related IR, increased plasma insulin, and multiple adipokines promote inflammation, endothelial dysfunction, and atherosclerosis, all of which contribute to hypertension and consequent cardiovascular events^3^,^4^,^5^,^6^. Regarding the choice of anti-hypertensive drugs for MetS, ACEIs, ARBs, CCBs, and MR blockers have beneficial effects on metabolic diseases^11^,^12^,^13^,^17^,^35^,^36^.

Large-animal models are of great value for pharmacological studies. NHPs are more similar to humans than other species with respect to drug metabolism, lipoprotein profiles, the development of cardiovascular diseases, and genetics. In addition, compared with humans, the advantages of our NHP model include drug-naive subjects, well-controlled laboratory conditions with respect to food composition and daily activity, and freedom from risk factors such as smoking and alcohol consumption. However, to date, there are no well-characterized MetS NHP models with hypertension. Thus, developing and characterizing NHP models with major diseases is essential for both basic and translational research as well as preclinical drug testing.

Spontaneously-occurring hypertension has been reported previously in NHPs^37^,^38^,^39^. But in those studies, relatively young animals (<10 years) were used and no metabolic index was investigated. Although previous reports from our lab^25^ and another group^24^ have shown that NHPs spontaneously develop MetS, hypertension was neither focused on nor deeply investigated in those studies. In the current study, we compared BP levels with repeated cuff measurement under ketamine anesthesia and recorded from freely-moving rhesus monkeys *via* telemetry. To our knowledge, this is the first time to set up a convenient method to identify a group of monkeys with naturally-developed hypertension and MetS. We performed detailed investigation of cardiovascular circadian rhythms (BP and HR) using data from elderly, freely-moving, control normotensive and MetS hypertensive rhesus monkeys. In addition, both the control normotensive and MetS hypertensive monkeys in our study were relatively old (control normotensive, 18.9 ± 1.1 and MetS hypertensive 18.4 ± 1.2 years), which mimics the condition in aging humans, who have a very high prevalence of both hypertension and MetS.

Using a telemetry system to continuously monitor BP in conscious, freely-moving monkeys, we validated our models with two anti-hypertension drugs, Amlodipine and Eplerenone, which have been shown to improve MetS with hypertension. In all of the prior studies of BP in monkeys^25^,^37^,^38^,^39^, BP was measured with a cuff in either anesthetized or restrained animals, which is unable to provide 24-h BP. The telemetry system enabled us to continuously and accurately measure BP in conscious, freely-moving monkeys. SBP and DBP showed significant correlations between cuff and telemetric measurements when measured simultaneously in the same animals. Cuff measurements may be more suitable for large scale, long-term studies. Telemetry is a better method to more accurately study the mechanisms of action and efficacy or safety of therapies.

Previous studies have suggested that elevated levels of aldosterone have adverse effects, including cardiovascular dysfunction, hypertension, IR, and organ damage^40^. When MetS hypertensive monkeys were chronically treated with Eplerenone, an MR blocker, BP decreased significantly. In addition, we found that it also reduced the blood concentrations of IL-6 and resistin; this could produce multiple beneficial effects on anti-hypertensive therapy including reducing inflammation, blocking the renin–angiotensin system (RAS), elevating vasorelaxation mediated by nitric oxide, alleviating IR, and preventing structural changes in the vasculature^33^. Furthermore, the potential function of MR blocker in reducing oxidative stress and L-type Ca channel activity in vascular smooth muscle cell might also contributes to the blood pressure lowering effect^41^. Here we provided 2 novel mechanisms of the protective effect of MR antagonists, including restoration of the impaired 24-h BP circadian rhythm and the decreased plasma AGE level.

Since the outcome of hypertension depends on not only the BP level, but also the circadian rhythm of BP^42^,^43^,^44^, we investigated this readout *via* telemetry in conscious monkeys. In patients with essential hypertension, losing the nocturnal fall in BP is associated with more serious organ damage than in patients whose BP falls during the night^45^, indicating the importance of proper evaluation of BP rhythms. Clinically, even with noninvasive measurement of 24-h BP, data collection with ambulatory blood pressure monitors over 24 h or longer can influence patient status, such as sleep quality and mood. These negatively impact assessment of the circadian BP rhythm. But with the implanted telemetry in our monkeys, there was no influence on routine life, further highlighting the uniqueness of the NHP hypertension model with telemetry as an ideal system for preclinical testing of the efficacy of anti-hypertensive drugs, especially newly-developed drugs.

AGEs are a heterogeneous group of reactive derivatives of nonenzymatic glucose-protein condensation reactions, as well as lipid and nucleic acids exposed to reducing sugars. AGEs have been proved to play a key role in the development of both diabetes and hypertension, together with their complications including myocardial infarction, heart failure, chronic renal failure and retinopathy^46^,^47^. And multiple studies have shown the correlation between the elevated blood AGE levels and these diseases^46^,^47^. Here for the first time, we identified that Eplerenone decreased plasma AGE concentration and is a novel inhibitor of AGE system. Some studies have evaluated the influence of RAS (especially ARBs) on the AGE-RAGE (the receptor of AGEs) axis, but with conflicting conclusions^48^,^49^,^50^,^51^. From our data, we provided the evidence that blockage of RAS decreases plasma AGEs in the NHP model with MetS and hypertension. Although in a previous study, inhibition of RAS reduced plasma sRAGE (soluble RAGE) level^52^, in our model, we did not find any change of plasma sRAGE concentration.

Although Eplerenone is a highly selective MR blocker, we can not exclude the possibility that its beneficial effect including the restoration of BP circadian rhythm and AGE levels may be also attributed to its off-target action in addition to the blockage of MR. It has been reported that Eplerenone may induce off-target effects through the androgen receptor, affecting hormone secretion and function^53^.

In summary, our study established a novel, naturally developed NHP model with both MetS and hypertension, offering a valuable tool for both mechanistic and translational cardiometabolic research. And with this model, we provided novel insights of the therapeutic mechanisms of MR blockers. Besides the previously reported beneficial effect, including BP decreasing and inflammation inhibition, Eplerenone also restores the 24-h BP circadian rhythm and reduced the plasma level of AGEs.

## Methods

### Animal and Housing

All of the experimental procedures were approved by the Institutional Animal Care and Use Committee of Peking University and were carried out in accord with the principles of laboratory animal care of the National Academy of Sciences/National Research Council. The rhesus monkeys (*Macaca mulatta*) were from our in-house cohort that was described in a previous report^25^, and were housed individually in cages in the Laboratory Animal Center of Peking University, with a 12-h light/dark cycle, a temperature range of 18–24 °C, and humidity of 40–70%. The monkeys had free access to water and were fed *ad libitum* with monkey chow (Beijing HFK Bio-Technology Co. Ltd, China), which contains 7–10% crude fat, 16–20% crude protein, and 55–65% crude carbohydrate.

### Blood Test and Cuff Blood Pressure Measurement

Body weight, waist and hip circumference, glucose tolerance, and blood biochemistry were measured every 6 months in all the in-house animals, following procedures described previously^25^. All of the data used in this study were from the average of the latest two tests. The cuff BP was measured using a mercury sphygmomanometer at 15–30 and 40–50 min after ketamine anesthesia (10 mg/kg, *i.m.*), and was calculated as the mean of the 2 measurements. HR was recorded on an electrocardiograph (MAC 1200ST, General Electric Co., Fairfield, CT, USA).

PRA was measured using an iodine (^125^I) angiotensin I radioimmunoassay kit (Beijing North Institute of Biological Technology, Beijing, China), plasma aldosterone was measured with an iodine (^125^I) aldosterone radioimmunoassay kit (Beijing North Institute of Biological Technology), and the data used for analysis was the average of two tests. Plasma resistin, IL-6, adiponectin, leptin, MCP-1, and TNF-α were assayed with Milliplex kits from Merck (Cat # HADK1MAG-61K and NHPMHMAG-45K). The ELISA kit for plasma AGEs and sRAGE was from Shanghai BlueGene Biotech CO.,LTD., China.

### Transmitter Implantation

To obtain continuous 24-h BP under freely-moving conditions in rhesus monkeys, BP was measured with an implantable telemetric transmitter from Data Sciences International, St Paul, MN, USA (DSI, model TL11M2-D70-PCT). The animals were fasted overnight and sedated with ketamine (10 mg/kg, *i.m.*), and the propofol (3–4 mg/kg) was injected through *i.v.* for intubation and maintained under inhaled Isoflurane (4% in oxygen for induction, 1.5–2.5% for maintenance) throughout the surgical procedure. Secure the animal in supine position on the surgery table, remove the body hair from all intended incision sites and sterilized with Povidone-iodine. Using a scalpel blade, a 4–5 cm midline incision was made through the skin and abdominal wall. Then the transmitter was inserted and sutured to the abdominal wall intraperitoneally. For arterial cannulation, a 2–3 cm incision was made through the skin over the femoral vessels. Then the femoral artery was isolated, and a trocar was used to tunnel subcutaneously from the femoral incision to the transmitter. The arterial BP catheter was passed through the trocar, and the catheter was introduced into the femoral artery until its tip was estimated to be in the terminal portion of the abdominal aorta. It was then secured in place by suture ties and the incision was closed. The animals were allowed to recover from surgery for at least 2 weeks before BP recording.

### Telemetry and Anti-hypertensive Drug Treatment

During telemetry, the animals were housed in individual recording cages and continuous recordings were made throughout the experiment periods. The BP data were analyzed as the mean of 1 or 24 h. For 24-h recordings, the mean value was from 07:00 to 07:00.

Data were recorded continuously for at least 4 days (baseline) before pharmacological treatment was initiated. All the animals used for telemetry had been trained for 2–3 years to cooperate for oral drug administration. The vehicle (orange juice) or drugs diluted in vehicle were delivered orally between 09:00 and 10:00 every day. Vehicle administration was also performed during baseline recording. Amlodipine besylate (Chempacific, Cat# 33369) was administered once a day between 09:00 and 10:00 for two days (1 mg/kg on day 1 and 3 mg/kg on day 2, *p.o.*). Eplerenone (30 mg/kg, *p.o.*) (Jiangxi Yuneng Medicine Chemical Co., Ltd., Ji’an, China, and provided by Merck for R&D use only) was administered once a day between 09:00 and 10:00 for 14 days.

### Rhythm Analysis

SBP, DBP and MBP, along with HR from each monkey were continuously collected as the average of every 20 seconds with the telemetry system, and assessed for the presence of a circadian rhythm by single cosinor analysis. Rhythm by group was assessed by population mean-cosinor analysis. A circadian rhythm was considered to be present if the model could be fitted (*F* value >3). The model reported two parameters. The MESOR is the median between the highest and lowest values of the fitted cosine. The amplitude is the distance between the MESOR and the highest (or lowest) value of the cosine curve. The amplitude represents the range of the rhythmic change. The cosinor analysis and graphs were performed in Mathemetica 9.0 (Wolfram Research, Inc., Champaign, IL, USA).

### Statistical Analysis

All data are expressed as mean ± SEM. Student’s t-test was used to evaluate differences between control and experiment groups. Paired t-test data were compared before and after medication. Data analysis was performed using the SPSS 18.0 software package (SPSS Inc., Chicago IL, USA). Two-sided *P* < 0.05 was used to denote statistical significance.

## Additional Information

**How to cite this article**: Zhang, Y. *et al.* Eplerenone restores 24-h blood pressure circadian rhythm and reduces advanced glycation end-products in rhesus macaques with spontaneous hypertensive metabolic syndrome. *Sci. Rep.* **6**, 23957; doi: 10.1038/srep23957 (2016).

## Supplementary Material

Supplementary Information

## Figures and Tables

**Table 1:** Metabolic and Anthropometric Parameters in Control Normotension and MetS Hypertension Monkeys.

	Control normotension (n = 17)	MetS hypertension (n = 16)	*P value*
Age, y	18.88 ± 1.10	18.38 ± 1.15	0.757
SBP, mmHg	116.03 ± 2.50	163.13 ± 5.49	<0.001
DBP, mmHg	74.04 ± 1.45	93.03 ± 3.05	<0.001
HR, bpm	127.94 ± 4.88	152.00 ± 6.45	0.005
WC, cm	38.06 ± 1.52	57.50 ± 3.68	<0.001
BW, kg	14.01 ± 0.50	22.01 ± 1.44	<0.001
TG, mmol/L	0.38 ± 0.03	0.75 ± 0.14	0.013
HDL-c, mmol/L	2.06 ± 0.14	1.72 ± 0.10	0.047
LDL-c, mmol/L	1.55 ± 0.32	1.44 ± 0.14	0.766
TC, mmol/L	3.63 ± 0.35	3.30 ± 0.15	0.411
FPG, mmol/L	4.05 ± 0.06	4.87 ± 0.23	0.001
Fasting insulin, mU/ml	12.54 ± 1.57	68.61 ± 22.00	0.013
HOMA-IR	2.25 ± 0.28	15.83 ± 5.83	0.023
CRP, mg/dl	0.12 ± 0.03	0.29 ± 0.07	0.027

Values are mean ± SEM.

MetS, metabolic syndrome; SBP, systolic blood pressure; DBP, diastolic blood pressure; HR, heart rate; WC, waist circumference; BW, body weight; TG, triglyceride; HDL-c, high-density lipoprotein; LDL-c, low-density lipoprotein cholesterol; TC, total cholesterol; FPG, fasting plasma glucose; HOMA-IR, homeostasis model assessment for insulin resistance. CRP, C-reactive protein.

**Table 2:** Amplitude of BP Circadian Rhythm in Control Normotension and MetS Hypertension Monkeys.

	Control normotension (n = 5)	MetS hypertension (n = 7)	*P value*
SBP(mmHg)	5.12 ± 2.86	2.61 ± 2.61	0.176
DBP(mmHg)	5.69 ± 1.40	2.19 ± 2.18	0.007
MBP(mmHg)	6.29 ± 1.49	3.25 ± 2.23	0.054
HR(bpm)	8.67 ± 3.57	7.63 ± 2.96	0.805

Values are mean ± SEM.

MetS, metabolic syndrome; SBP, systolic blood pressure; DBP, diastolic blood pressure; MBP, mean blood pressure; HR, heart rate.

**Table 3:** Amplitude of BP Circadian Rhythm before and after Eplerenone treatment in MetS Hypertension Monkeys.

	Baseline	Eplerenone	*P value*
SBP(mmHg)	2.07 ± 2.88	4.00 ± 1.57	0.470
DBP(mmHg)	2.89 ± 1.85	3.65 ± 1.10	0.099
MBP(mmHg)	3.93 ± 1.82	4.89 ± 1.05	0.004
HR(bpm)	6.86 ± 2.25	8.64 ± 1.27	0.129

Values are mean ± SEM. n = 7.

MetS, metabolic syndrome; SBP, systolic blood pressure; DBP, diastolic blood pressure; MBP, mean blood pressure; HR, heart rate.
