# Supplementary material for: Eplerenone restores 24-h blood pressure circadian rhythm and reduces advanced glycation end-products in rhesus macaques with spontaneous hypertensive metabolic syndrome
Source: Sci Rep. 2016 Apr 1;6:23957. doi: 10.1038/srep23957 (PMC4817044; doi:10.1038/srep23957)
Supplement: Supplementary Information [file srep23957-s1.pdf]

Title: **Eplerenone restores 24-h blood pressure circadian rhythm and reduces advanced glycation end-products in rhesus macaques with spontaneous hypertensive metabolic syndrome**

Yan Zhang[1,3],Wen Zheng[1,3], Yuli Liu[1,3], Jue Wang[1,3], Ying Peng[4], Haibao Shang[1,3], Ning Hou[1,3], Xiaomin Hu[1,3], Yi Ding[1,3], Yao Xiao[1,3], Can Wang[1,3], Fanxin Zeng[1,3], Jiaming Mao[1,3], Jun Zhang[1,3], Dongwei Ma[1,3], Xueting Sun[1,3], Chuanyun Li[1,3], Rui-Ping Xiao[1,2,3]*, Xiuqin Zhang[1,3]*

[1]State Key Laboratory of Membrane Biology, Institute of Molecular Medicine, Peking University, Beijing 100871, China.
[2]Peking-Tsinghua Center for Life Sciences, Beijing 100871, China.
[3]Beijing Key Laboratory of Cardiometabolic Molecular Medicine, Peking University, Beijing 100871, China.
[4]Department of Surgery, Third Affiliated Hospital of Peking University, Beijing 100871, China.

*Corresponding author:

Xiuqin Zhang, M.D., Ph.D.
Institute of Molecular Medicine,
Peking University, Beijing 100871, China.
E-Mail: zhangxq@pku.edu.cn
Tel: (086)-10-6275-3420.
Fax: (086)-10-6276-7143.

Rui-Ping Xiao, M.D., Ph.D.
Institute of Molecular Medicine,
Peking University, Beijing 100871, China.
E-Mail: xiaor@pku.edu.cn
Tel: (086)-10-6275-7243.
Fax: (086)-10-6276-7143.

Supplemental Material

SUPPLEMENTAL FIGURES

Supplemental Fig. 1 Correlation between telemetric and cuff BP. Systolic blood pressure (**a**) and diastolic blood pressure (**b**) of 12 monkeys with telemetric implants (5 control normotensive and 7 metabolic syndrome hypertensive) measured simultaneously with a mercury sphygmomanometer (cuff) and telemetry 15 min after anesthesia with ketamine (10 mg/kg, *i.m.*).

Supplemental Fig. 2 Plasma concentrations of potassium (**a**), TCh (**b**), TG (**c**), ALT and AST (**d**), urea (**e**), and creatinine (**f**) of MetS hypertensive (n = 7) monkeys at baseline, treated with Eplerenone for 14 days (30 mg/kg/day, *p.o.*, once a day) and washed out for 13 days. Data are expressed as mean ± SEM. MetS, metabolic syndrome; TCh, total cholesterol, TG, Triglyceride; ALT, aspartate aminotransferase; AST, alanine aminotransferase.

Supplemental Fig. 3 Plasma concentrations of adiponectin (**a**), leptin (**b**), MCP-1 (**c**), and TNF-α (**d**) of the MetS hypertensive monkeys at baseline and after 14 days of Eplerenone treatment. Data are expressed as mean ± SEM. MCP-1, monocyte chemoattractant protein-1; TNF-α, tumor necrosis factor α.

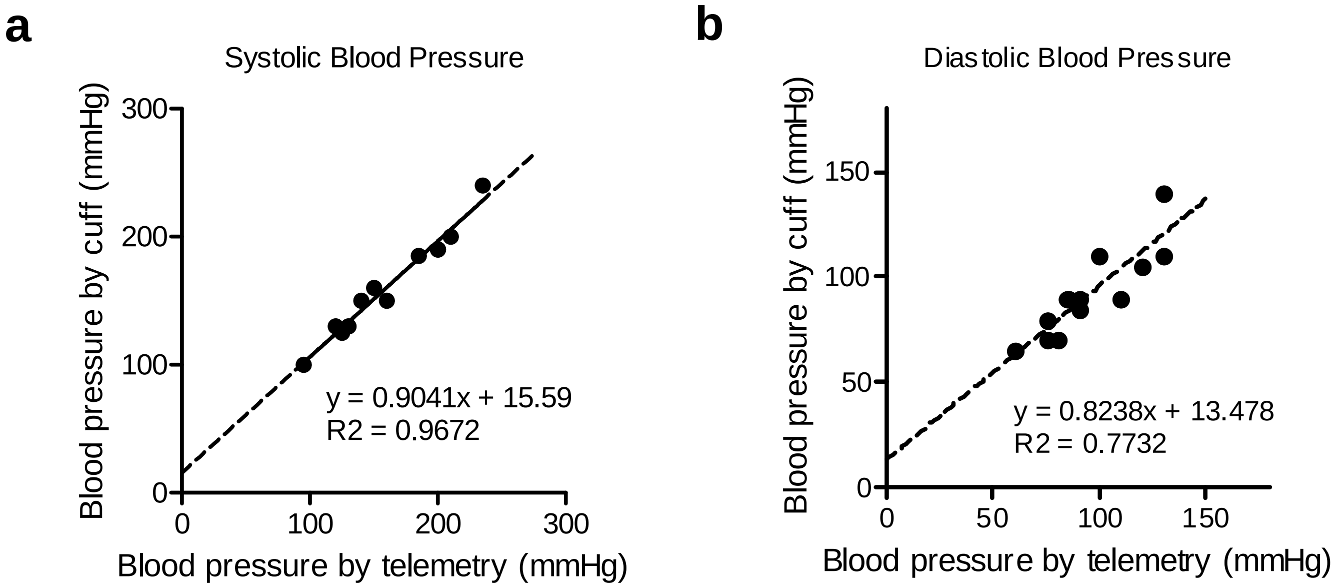

Supplemental Figure 1

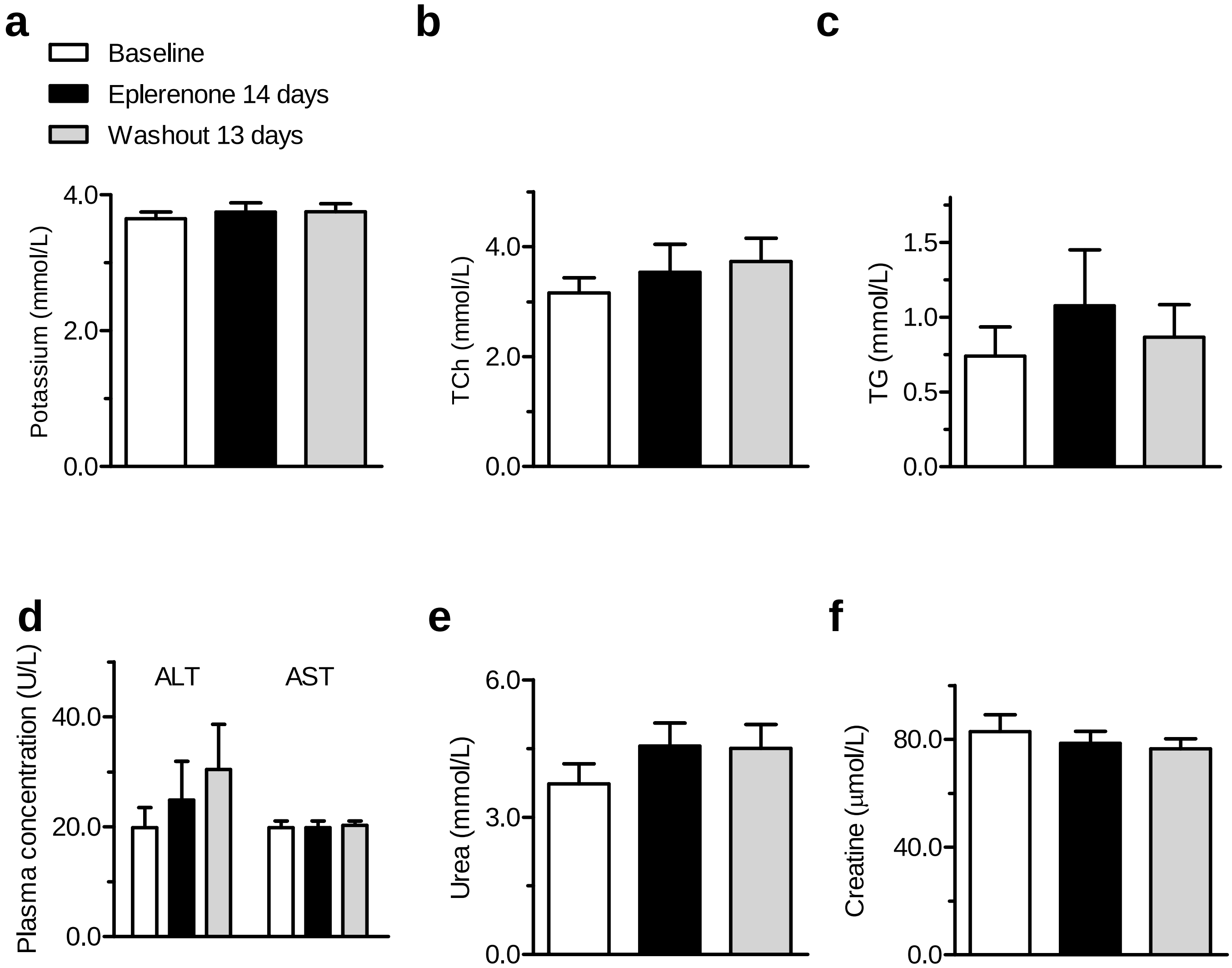

Supplemental Figure 2

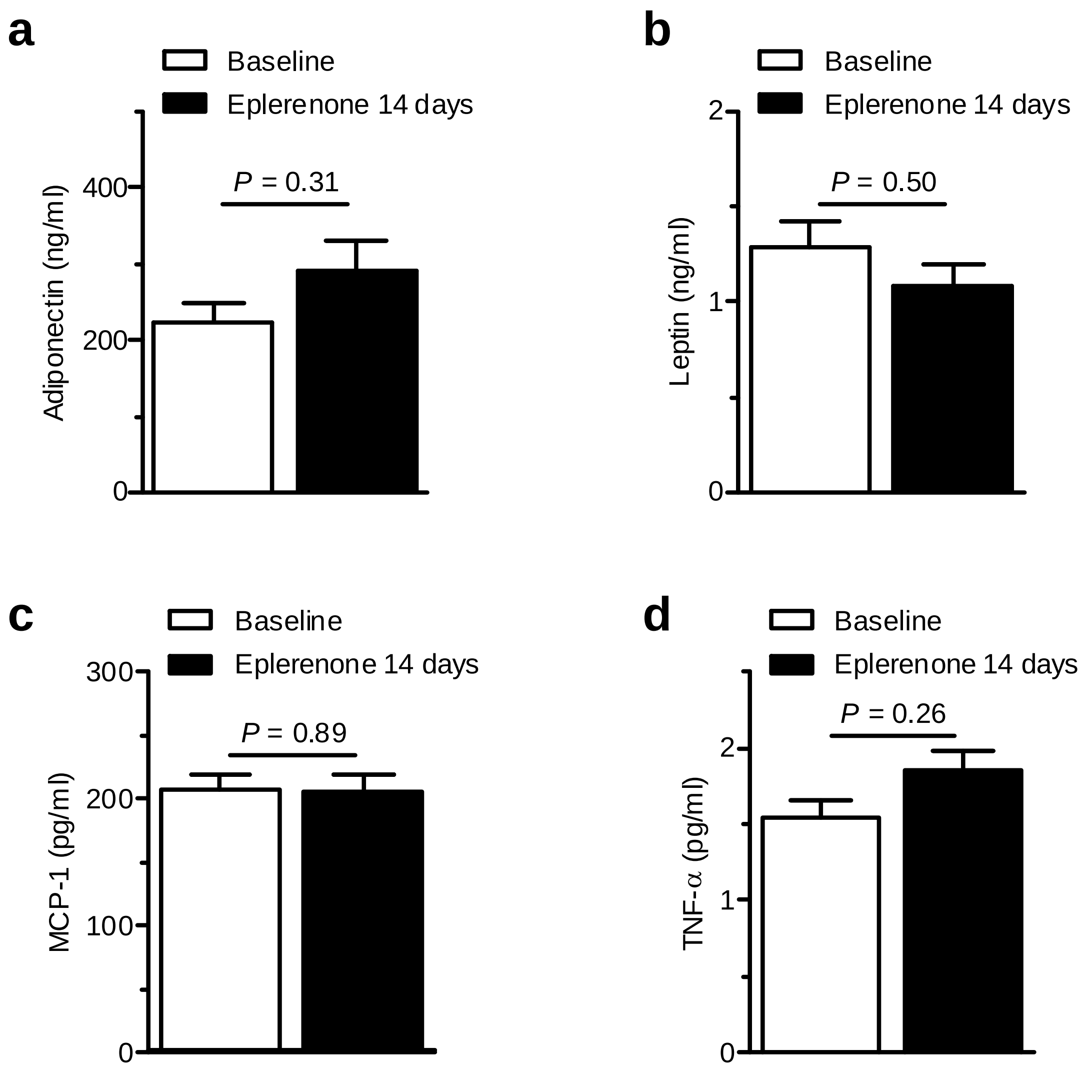

Supplemental Figure 3

Supplemental Table 1. *F* value of the BP Circadian Rhythm in Control Normotension and MetS Hypertension Monkeys

Group	Animal ID#	SBP	DBP	MBP	HR
	1	> 3	> 3	> 3	0.361
	2	> 3	> 3	> 3	> 3
Control normotension	3	> 3	> 3	> 3	> 3
	4	> 3	> 3	> 3	1.036
	5	> 3	> 3	> 3	> 3
	1	2.562	> 3	> 3	> 3
	2	> 3	> 3	> 3	> 3
	3	> 3	> 3	> 3	> 3
MetS hypertension	4	> 3	1.427	> 3	> 3
	5	> 3	> 3	> 3	> 3
	6	2.169	> 3	> 3	> 3
	7	> 3	> 3	> 3	> 3

MetS indicates metabolic syndrome; SBP, systolic blood pressure; DBP, diastolic blood pressure; MBP, mean blood pressure; HR, heart rate.

Supplemental Table 2. *F* value of the BP Circadian Rhythm before and after Eplerenone treatment in MetS Hypertension Monkeys

Group	Animal ID#	SBP	DBP	MBP	HR
	1	> 3	1.925	> 3	0.361
	2	> 3	> 3	> 3	> 3
	3	1.039	> 3	> 3	> 3
Baseline	4	1.324	> 3	> 3	> 3
	5	> 3	> 3	> 3	> 3
	6	1.609	> 3	> 3	> 3
	7	> 3	2.285	> 3	> 3
	1	> 3	> 3	> 3	> 3
	2	> 3	> 3	> 3	> 3
	3	> 3	> 3	> 3	> 3
Eplerenone	4	0.390	> 3	> 3	> 3
	5	> 3	> 3	> 3	> 3
	6	0.617	> 3	> 3	> 3
	7	> 3	> 3	> 3	> 3

MetS indicates metabolic syndrome; SBP, systolic blood pressure; DBP, diastolic blood pressure; MBP, mean blood pressure; HR, heart rate.
